# Design and evaluation of 3D-printed auxetic structures coated by CWPU/graphene as strain sensor

**DOI:** 10.1038/s41598-022-11540-x

**Published:** 2022-05-11

**Authors:** Hyeong Yeol Choi, Eun Joo Shin, Sun Hee Lee

**Affiliations:** 1 Department of Fashion Design, Dong-A University, Busan, 49315 Republic of Korea; 2 Department of Organic Materials and Polymer Engineering, Dong-A University, Busan, 49315 Republic of Korea

**Keywords:** Materials science, Composites

## Abstract

A strain sensor characterized by elasticity has recently been studied in various ways to be applied to monitoring humans or robots. Here, 4 types of 3D-printed auxetic lattice structures using thermoplastic polyurethane as raw material were characterized: truss and honeycomb with positive Poisson's ratio and chiral truss and re-entrant with negative Poisson's ratio. Each structure was fabricated as a flexible and stable strain sensor by coating graphene through a dip-coating process. The fabricated auxetic structures have excellent strength, flexibility, and electrical conductivity desirable for a strain sensor and detect a constant change in resistance at a given strain. The 3D-printed auxetic lattice 4 type structures coated with CWPU/Graphene suggest potential applications of multifunctional strain sensors under deformation.

## Introduction

Strain sensors are used to detect deformation or structural changes occurring in the surrounding environment as well as internal activities in human bodies. A multitude of studies have been conducted to develop efficient strain sensors, and materials that respond to structural changes and exhibit such change have been mainly explored^1–3^. Strain sensors require great flexibility and play an important role in a variety of applications such as electronic skin^4,5^ and soft robots^6,7^.

3D printing is a method of manufacturing an object with a desired shape through the process of continuously depositing designed materials under computer control. It has the advantage of being able to easily create the shape of a complex model that overcomes the limitations of the existing cutting process method^8–10^. Ever-evolving new technologies are being reported, starting with the stereo lithography-based production of Charles Hull's solid imaging process technology, which has made the greatest contribution to the advancement of 3D printing^11^. Numerous studies are underway on 3D printers, including various prototypes^12–14^, output processes and product completeness^15–18^, as well as strain sensors^19^, and innovative developments are being made to apply 3D printers more efficiently and inexpensively in various fields of both industry and academia, such as engineering ^20,21^, construction^22,23^, aviation^24,25^, fashion^26–28^, and biology field^29–31^.

Among the versatile 3D printing materials mentioned above, thermoplastic polyurethane (TPU) is a very attractive material. TPU is a low-cost material, highly suitable for 3D printing^32^, has the advantages of both the rubber and rigid thermoplastic resin, so it can be easily plastically deformed under the influence of heat, does not stick like rubber, and has high elasticity because it is manufactured with excellent adhesion. These outstanding properties have the potential to develop strain sensors that respond to stress and strain as well as low operating voltages and to develop multifunctional sensory materials.

Graphene, a two-dimensional hexagonal material that will be responsible for the electrical signal of the strain sensor, is useful for detecting tensile strain with high sensitivity in strain sensors that require flexibility and is being intensively applied to monitoring human movement^33,34^. This is due to its exceptional conductivity and mechanical strength. However, in order for graphene to optimally detect electrical signals from sensors, it must compensate for its lack of flexibility. Waterborne polyurethane (WPU) is one of the best materials for dispersing graphene. It is friendly to the human body and the environment in terms of odor and toxicity of organic solvents and has excellent viscosity control and film-forming properties^35^. Moreover, several studies on a WPU-based coating solution using graphene as a filler have been conducted in various fields. This is because, as a composite material, the flexibility and elasticity of WPU and the durability of graphene are improved, and at the same time, graphene forms a network as a filler, enhancing electrical, mechanical, and thermal properties^36–38^.

Caster-oil-based waterborne polyurethane (CWPU) can synthesize WPU from renewable raw materials such as vegetable oil-based materials, which are non-edible, accordingly no need to compete with edible oils. A major challenge in the development of CWPU is increasing the use of biomass to replace fossil fuels due to economic and environmental concerns. However, the performance of CWPU is not inferior to that of solvent-borne polyurethane in terms of mechanical properties, flexibility, and mobility, so its applications are diverse^39^.

In this study, TPUs of four different structures were printed through a fused deposition modeling (FDM) 3D printing method and used as strain sensors, and FDM is one of the safest, simple, and most widely used 3D printing approaches. First, a chiral truss (CT) and re-entrant (RE) structure were used as auxetic lattice materials with a negative Poisson's ratio, a new type of metamaterial that expands transversely in response to axial stretch. For comparison, a truss (TR) and honeycomb (HN) structure were analyzed as two types of regular structures with a positive Poisson's ratio. Also, in this study, a dip-coating method using graphene was used, which is one of the easy and efficient methods of efficiently imparting electrical conductivity to materials^40,41^. Therefore, all structures were designed to coat the surface by applying the dip-coating method with CWPU/Graphene solution. The four structures coated with up to 5 layers of graphene were analyzed as sensors that respond to strain centered on Poisson's ratio by elongation.

## Methods

### Materials

In order to 3D print the auxetic lattice structures, NinjaFlex (Ninjatek, Fenner Inc., USA and Shenzhen Esun Industrial Co., Ltd, China), a TPU-based filament, with a diameter of 1.75 mm and a hardness of 85A was used. A CFDM 3D printer (Blackbelt 3D B.B., Netherland), which uses a continuous fused deposition modeling, with a nozzle diameter of 0.6 mm was used.

The anionic castor oil-based waterborne polyurethane was prepared through the following steps: (1) raw material mixing step of adding isophorone diisocyanate (IPDI) dropwise to a mixture of castor oil and dimethylolbutanoic acid (DMBA) while continuously stirring, (2) synthesizing urethane step in which urethane synthesized while lowering the viscosity of the pre-polymer with acetone and, (3) neutralization step of the carboxylic group of DMBA with triethylamine, (4) forming water-borne polyurethane (WPU) particles step by dropping distilled water into prepolymer solution, (5) acetone removal step in the WPU aqueous solution. The content of natural substances in the prepared WPU is about 50%, the equivalent ratio of OH (castor oil)/NCO (IPDI)/OH (DMBA) is 1:2.2: 1.19, and the content of the hard segment of the WPU is 59.4%. The particle size is (180 ± 10) nm, and the zeta potential is (−30 ± 2) mV.

The graphene (GNP-UC) has 3–10 layers, thicknesses of 5–6nm, and lengths of 5–10 mm, and was purchased from Carbon Nanotech Co., Ltd. (Korea).

### Modelling of 3D printed pattern of auxetic lattice 4-structure

Table 1 shows 3D modeling and printed products for each pattern of 3D-printed auxetic lattice 4 type structures. To prepare the TR, HN, CT, and RE auxetic lattice materials, we designed a single repeating unit of 10 mm (length) × 10 mm (width) with a 2 mm line width (17 mm × 17 mm for the CT). The final model with a size of 120 mm × 1200 mm was created by arranging 12 repeating units horizontally and 120 repeating units vertically. 3D modeling was performed with a thickness of 1 mm using the 123D Design program of Autodesk Co., Ltd, (Korea). During printing work, the nozzle temperature, printing angle, printing speed, and infill density were set at 240 °C, 15°, 15 mm/s, and 100%, respectively. It was transformed into a g-code file to be printed. The obtained 4 type structures were named TR, HN, CT, and RE, and all the structures were cut to 50 mm × 50 mm after removing the edges to maintain the auxetic structure.

**Table 1:**
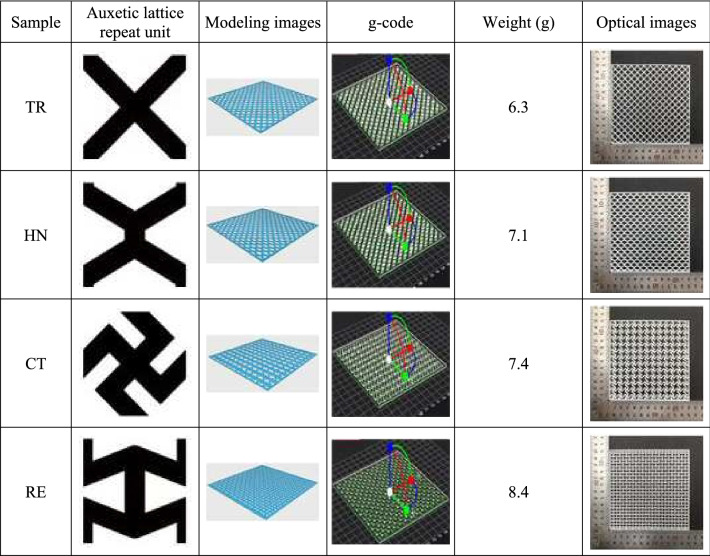
3D modeling and output of 3D-printed auxetic lattice 4 type structures.

### Preparation for coating of CWPU/Graphene water-based solution on 4-structure and stain sensing measurement

**Figure 1:**
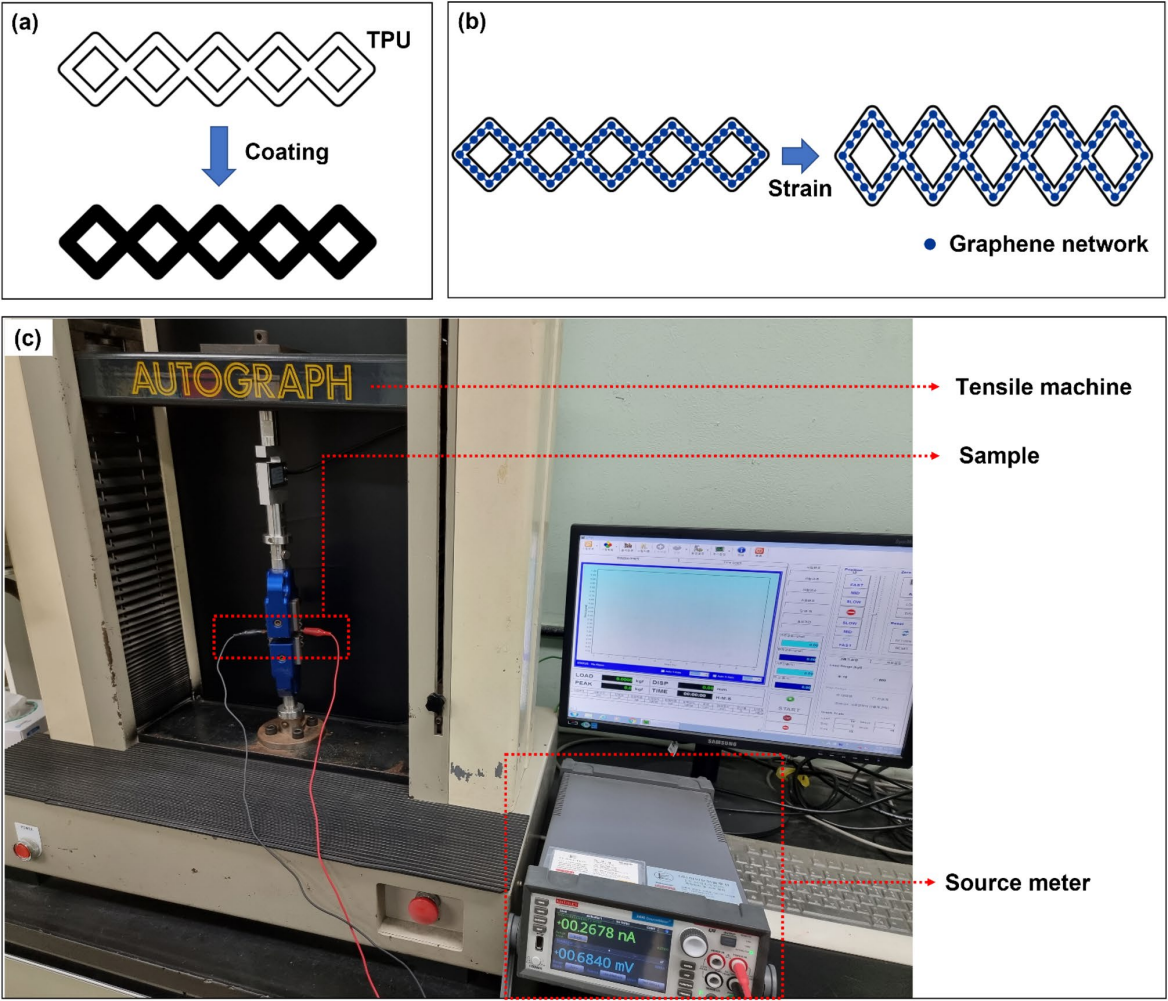
The overall the experimental setup and the detection mechanism (**a**) characterization of auxetic structure (**b**) scheme of sensing mechanism of graphene coated auxetic structure by strain (**c**) tensile test system of strain sensing.

A coating solution for the 3D-printed auxetic lattice 4 type structures was prepared as follows. 8.0 wt% graphene was added to a 30.0 wt% CWPU solution dispersed in water. The prepared CWPU/Graphene water-based solution was stirred at least 24 hours, followed by 1 hour of sonication, before being used as the coating solution. We continued to stir the solution before use. The 4 structures printed by the 3D printer were dip-coated with the coating solution prepared above and dried in an oven at 40°C for 1 hour before use. One hour of drying was considered the end of one cycle of coating, and the coating was repeated 5 times. Figure 1 shows the overall abstract images of our study, the experimental setup, and the detection mechanism. Through the coating process mentioned above in Fig. 1a, the TPU structure is dip-coated, and the surface becomes black by graphene. A characteristic of meta-structures is that in all structures there are holes according to the designed shape. Figure 1b shows the sensing mechanism. For the strain sensor, it converts the strain based on the flexibility of the TPU. With the resistance change dependent on the movement of the graphene network. As the sample is stretched, the graphene particles separate or become more distant, increasing the resistance value. Figure 1c is the configuration of the equipment for the sensing measurement of the strain sensor. The extension type tensile testing machine holds and stretches the sample. At the same time, each structure detects strain based on the electrical signal and flexibility of graphene. At the same time, the detected signal is measured with a source meter, and the signal is interpreted and analyzed through a connected computer.

### Characterization

The mechanical properties of 3D-printed auxetic lattice 4 type structures were characterized using a constant rate of extension type tensile testing machine (AGS-500D, Shimadzu, Japan). The distance between the clamps gripping the sample in the machine direction (MD) was set to be the length of the single repeating unit of each structure: 10 mm for the TR, HN, and RE, and 17 mm for the CT. The maximum elongation based on the gauge length was tested up to 60%, and the initial modulus, tensile strength, and toughness were analyzed.

The electrical properties of the coated auxetic lattice 4 type structures were characterized using a source meter (2450 Source Meter, Keithley Instruments). To investigate the efficiency of increasing electrical performance by coating with graphene solution and to explore the electrical properties applied in MD and counter direction (CD), the current–voltage curves of the structure for each coating cycle were measured in MD and CD and analyzed. The electrical conductivity was calculated using the following Eq. (1):

1 $$\sigma =\frac{1}{\rho }=\frac{1}{R}\times \frac{L}{A}$$

where *σ* is the electrical conductivity, *ρ* is the resistivity, *R* is the resistance, *A* is the cross-sectional area of the sample, and *L* is the length of the sample. Here, the cross-sectional area of the sample includes space, but meta-structure is defined as a structure that includes space, so it is assumed to be a rectangle. The practical conductivity of the coating was calculated only by the actual increment of the coating.

The relative resistance change was calculated by grab the sample at the constant rate of extension machine as above mentioned, connecting electrodes to both ends, and applying 0.01V through the source meter. The strain was elongated by 1, 3, 5, 10, 15, 20, 25, and 30% against the grab distance, respectively. The relative resistance change (*R*_*r*_) was calculated using the following Eq. (2):

2 $$Rr=(R-{R}_{0})/{R}_{0}\times 100\%$$

where *R* is the resistance during elongation and *R*_*0*_ is the initial resistance. The image of each strain was taken with a camera (HDR-CX550, Sony, Japan) to measure the change in relative resistance. Then, the longitudinal and transverse extension points were identified to calculate the strains in each direction, and the Poisson's ratio was calculated using the following Eq. (3):

3 $$\nu =-{\varepsilon }_{t}/{\varepsilon }_{l}$$

where *ν* is the Poisson's ratio, *ε*_*t*_ is the transverse strain, and *ε*_*l*_ is the longitudinal strain.

The gauge factor was calculated as the strain and the rate of change of the relative resistance for the 4 type structures based on the following Eq. (4):

4 $$GF= \frac{(R-{R}_{0})/{R}_{0}}{\varepsilon }$$

where *R* is the resistance during elongation and *R*_0_ is the initial resistance, and *ε* is the strain.

## Results and discussion

### Morphology and weight and thickness increase rate

Table 2 shows the images of the 3D-printed auxetic lattice 4 type structures taken in the dip-coating process where CWPU/Graphene water-based solution was used, and the coating was conducted up to 5 times. In all the structures, the color change was clearly observed as the coating cycle progressed, and, accordingly, the weight and thickness increased linearly. The dip-coating process using the graphene coating solution is efficient and has a great influence on increasing electrical conductivity for electrical signal sensing^42^. In particular, the number of dip-coatings has a clear correlation with physical durability and electrical conductivity and varies from a few to as many as 20 times depending on the study^43,44^. In our previous study, we have demonstrated that 5 dip-coating using the WPU and graphene composite solution is appropriate and efficient and applied in the present study^36^.

**Table 2:**
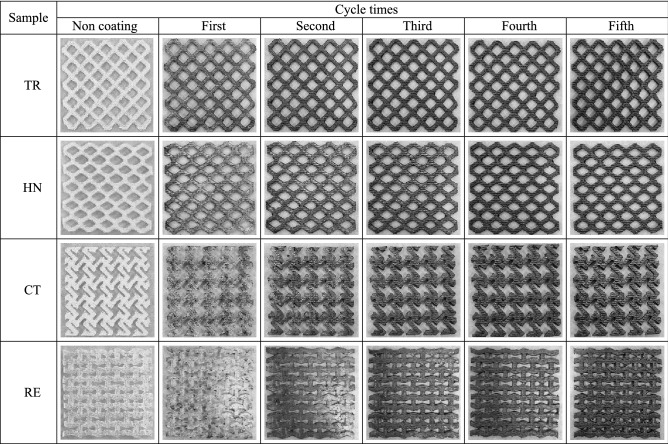
Optical images for up to 5th cycle coatings of auxetic lattice 4 type structures.

**Figure 2:**
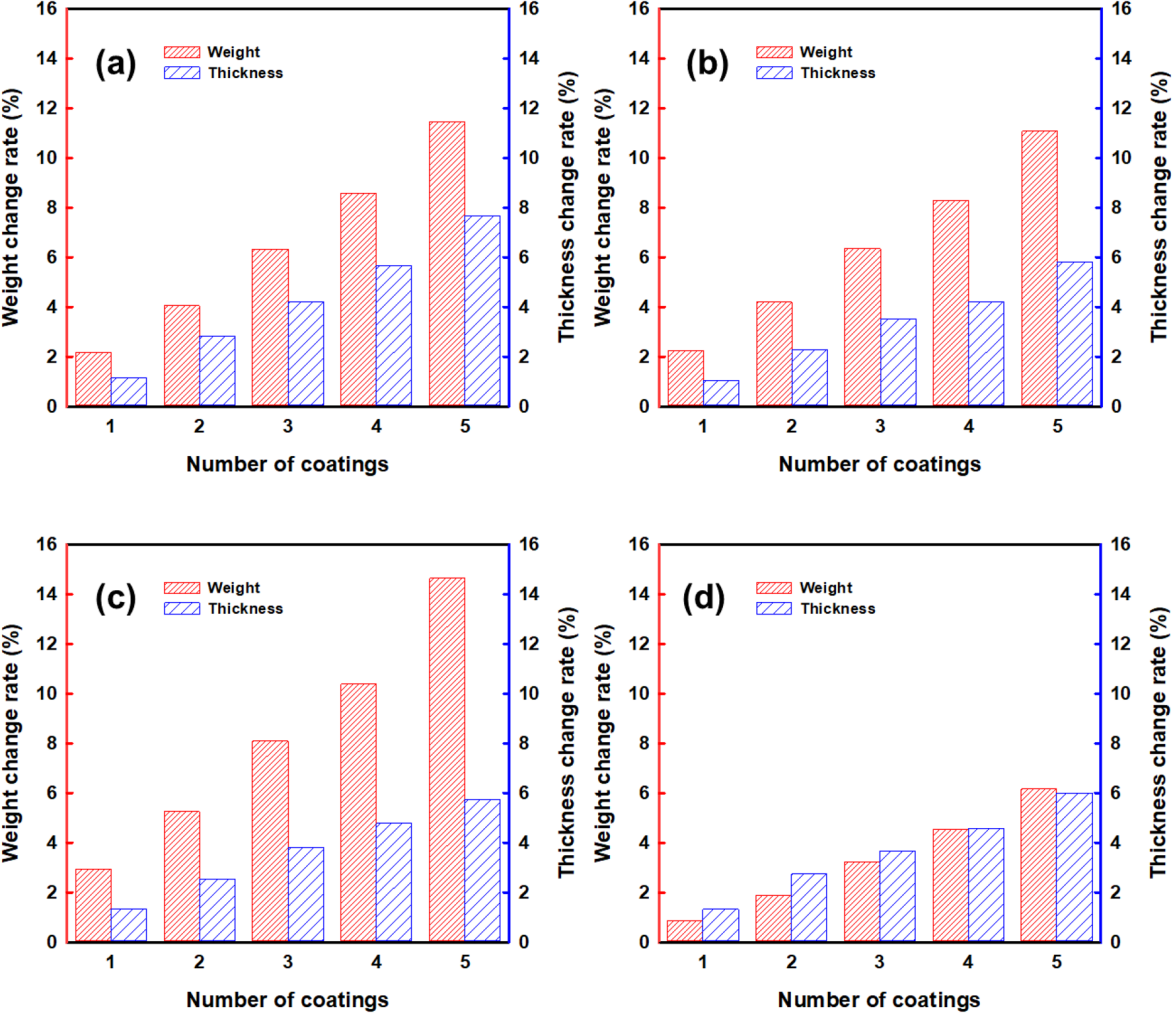
Weight and thickness increase rate of auxetic lattice 4 type structures: (**a**) TR, (**b**) HN, (**c**) CT, and (**d**) RE.

Figure 2 shows the change rate in weight and thickness of the 4 type structures when the CWPU/Graphene water-based solution was coated 5 times. The CWPU/Graphene coating layer increased in weight and thickness linearly in all structures. This indicates that the coating proceeded smoothly and that a coating layer is physically and gradually formed on the outer layer of the TPU after the CWPU/Graphene solution is dried in the water dispersion state. Particularly, RE has the highest initial weight since it has a denser repeating unit than other structures due to its compact structure. Therefore, the rate of increase in weight is measured to be the lowest.

### Mechanical property

**Figure 3:**
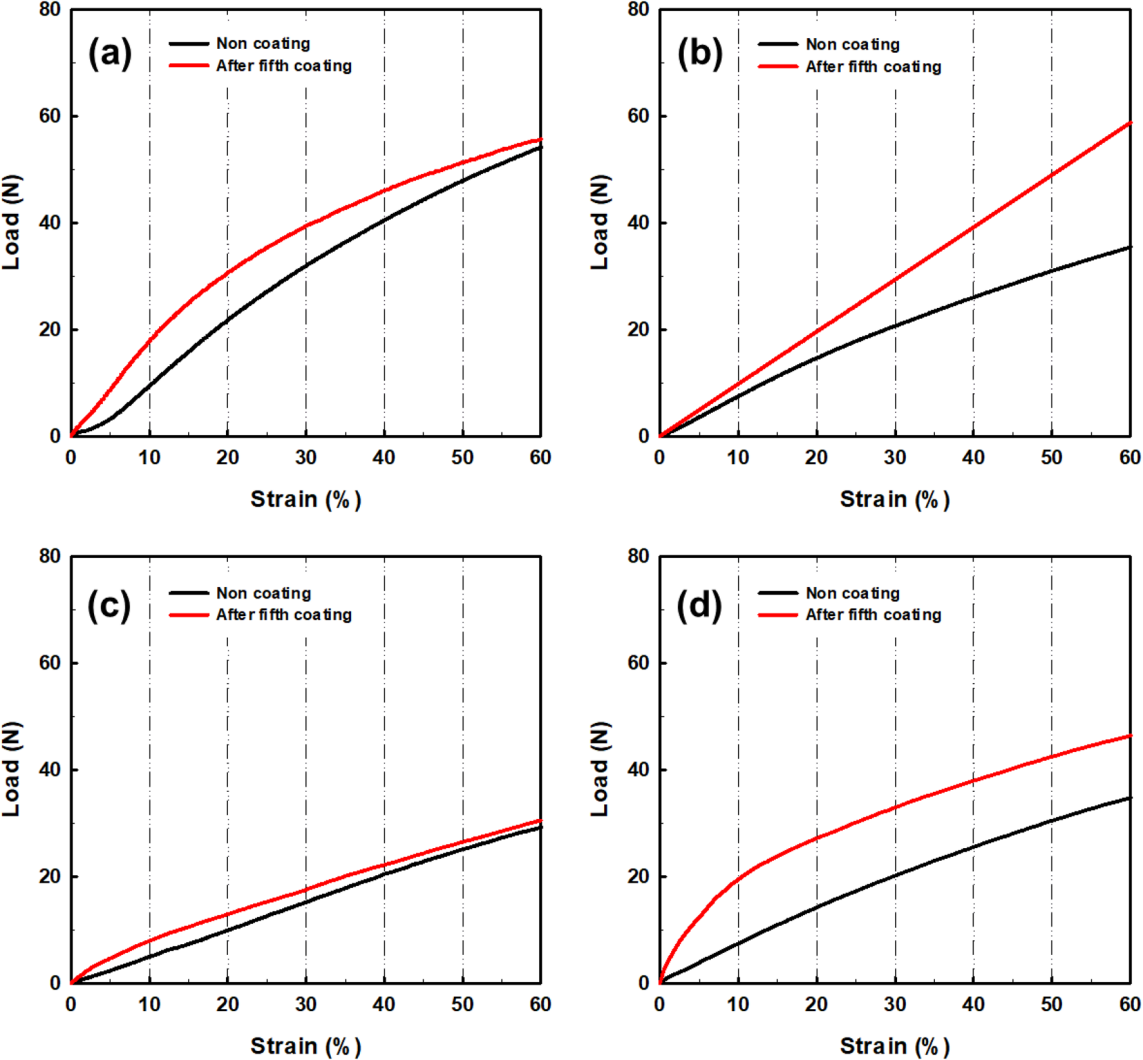
Stress–strain curves of compared to before and after fifth coated auxetic lattice 4 type structures: (**a**) TR, (**b**) HN, (**c**) CT, and (**d**) RE.

Figure 3 and Table 3 compare the mechanical properties before and after the coating to analyze the mechanical strength and stretchability of 3D-printed auxetic lattice 4 type structures. Figure 2a–d shows the tensile stress–strain curves before and after the coating when elongated to 60% for the 4 type structures. Overall, the mechanical strength appeared to increase as the coating cycle progressed. In general, although there is a difference depending on the content, coating single graphene or composite solution imparts an appropriate amount of mechanical strength to the applied matrix^45^. Also, as shown in Table 3, the coating increases the initial modulus at 1% strain and toughness at 60% strain, regardless of the structure. This shows that coating of auxetic TPU with CWPU/Graphene water-based solution 5 times increases all mechanical properties without degrading strength, resulting in stable coating efficiency.

**Table 3:** Mechanical properties of compared with before and after fifth coated auxetic lattice 4 type structures at 60% strain.

Sample	Load (N)	Initial modulus (N)	Toughness (J)
TR	54.2	0.72	0.178
TR after coating	55.5	1.96	0.214
HN	35.6	0.74	0.119
HN after coating	58.8	1.15	0.176
CT	29.2	0.50	0.090
CT after coating	30.5	1.21	0.103
RE	34.9	0.99	0.163
RE after coating	46.5	4.00	0.263

### Electrical property

**Figure 4:**
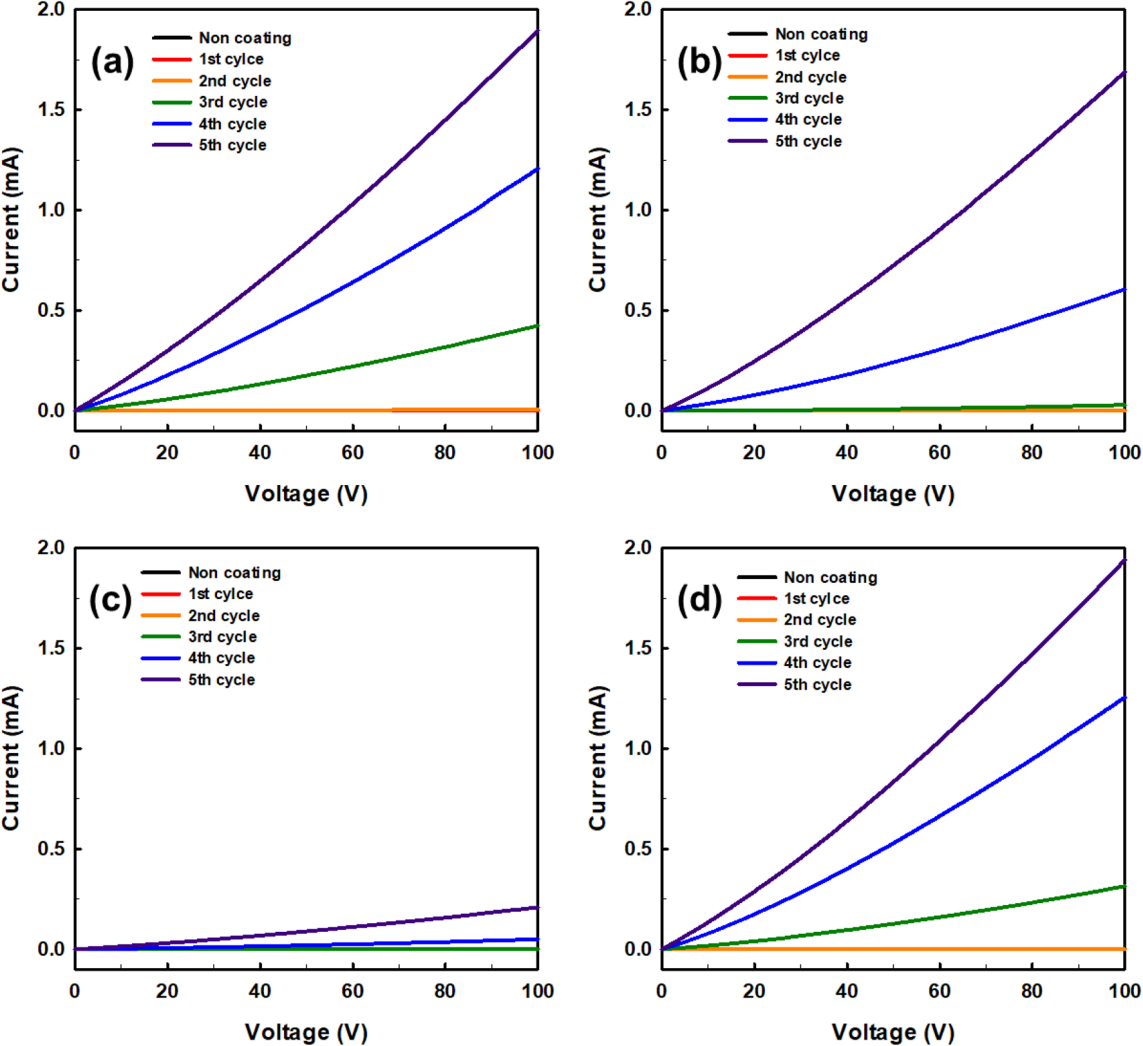
Current–voltage (I–V) curves of compared with before and every coating cycle of auxetic lattice 4 type structures for CD direction: (**a**) TR, (**b**) HN, (**c**) CT, and (**d**) RE.

**Figure 5:**
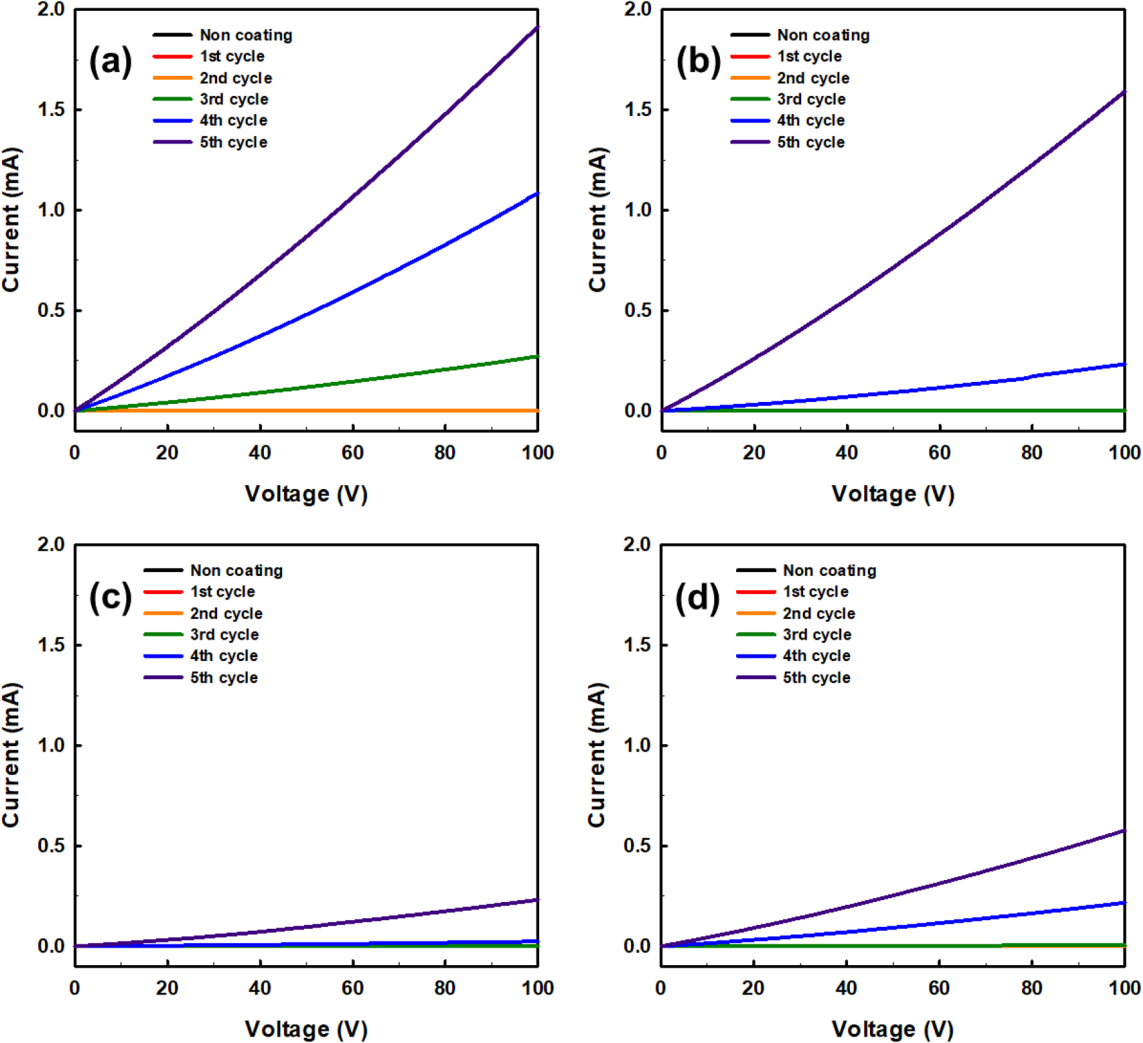
Current–voltage (I–V) curves of compared with before and every coating cycle of auxetic lattice 4 type structures for MD direction: (**a**) TR, (**b**) HN, (**c**) CT, and (**d**) RE.

In order to analyze the electrical properties of the 3D-printed auxetic lattice 4 type structures that serve as the strain sensors after coating, the current–voltage (IV) characteristics in the CD and MD were measured for each coating cycle as shown in Figs. 4 and 5. The sensing range of current and voltage for sensing under strain should conform to Ohm's law for reliable results of the sensor^46^. As can be seen from Fig. 4a–d, the current increases linearly with the applied voltage as the coating progress in all 4 type structures. In general, it was confirmed that at least fourth cycles of the coating were required for the electrical current to flow to all structures. This is presumably because a sufficient network between graphene having electrical conductivity may not be formed until after two or three cycles of coatings. Also, this result is consistent with the time when the overall color turns gradually black, as shown in Table 2. In particular, all CT structures have relatively disadvantageous paths through which current can flow compared to other structures due to the inherent complexity of the structures. In Fig. 5a–d, the current flowing through the MD of the 4 type structures was compared with the current flowing through the CD. As a result, it was found that current flows smoothly in all structures regardless of direction. However, in the case of the RE structure, the slopes of the I-V curves in the MD and CD are different, unlike other structures, indicating that the pathway through which the current flows is related to the structural characteristics of each structure. Unlike TR, CT, and HN, which have little or no structural difference in their pathways in the MD and CD, RE has a huge structural difference, implying that such structural characteristics should be considered when using RE as a strain sensor.

Table 4 summarizes the apparent and practical conductivity according to the actual coating thickness by calculating the electrical conductivity after obtaining the surface area of the 3D-printed auxetic lattice 4 type structures using Image J. The apparent conductivity of the uncoated structures was measured within a range of ~ 10^–11^−10^–12^ S/cm digits of the nonconductor level. The electrical conductivity increased with each cycle of coating, and after 5 cycles of the coating, each structure exhibited an electrical conductivity ranging from ~ 9.08 × 10^–06^ to ~ 1.96 × 10^–03^ S/cm. In addition, the electrical conductivity with coating thickness actually increases significantly from 3.23 × 10^–04^ to 1.96 × 10^–03^ S/cm. The electrical conductivity of each sample coated 5 times is sufficient to detect currents as a strain sensor, even including voids created due to the structural properties of the 4 type structures.

**Table 4:** Electrical properties of compared with before and every coating cycle of auxetic lattice 4 type structures.

Sample		CD-apparent conductivity (S/cm)	CD-practical conductivity (S/cm)	MD-apparent conductivity (S/cm)	MD-practical conductivity (S/cm)
TR	Non coating	5.34 × 10^−11^	–	7.69 × 10^−11^	–
	1st cycle	7.61 × 10^−11^	5.61 × 10^−09^	2.06 × 10^−10^	1.52 × 10^−08^
	2nd cycle	1.11 × 10^−07^	5.53 × 10^−06^	1.93 × 10^−10^	9.58 × 10^−09^
	3rd cycle	1.55 × 10^−05^	4.28 × 10^−04^	1.01 × 10^−05^	2.79 × 10^−04^
	4th cycle	3.98 × 10^−05^	9.47 × 10^−04^	4.27 × 10^−05^	1.02 × 10^−03^
	5th cycle	1.17 × 10^−04^	2.19 × 10^−03^	7.86 × 10^−05^	1.47 × 10^−03^
HN	Non coating	5.24 × 10^−11^	–	3.97 × 10^−11^	–
	1st cycle	8.86 × 10^−10^	1.95 × 10^−08^	3.48 × 10^−11^	6.21 × 10^−09^
	2nd cycle	2.19 × 10^−10^	1.64 × 10^−08^	5.71 × 10^−10^	3.43 × 10^−09^
	3rd cycle	1.78 × 10^−07^	8.14 × 10^−06^	8.65 × 10^−09^	3.15 × 10^−07^
	4th cycle	1.32 × 10^−05^	4.34 × 10^−04^	6.84 × 10^−06^	1.80 × 10^−04^
	5th cycle	4.74 × 10^−05^	1.24 × 10^−03^	2.09 × 10^−05^	4.33 × 10^−04^
CT	Non coating	4.97 × 10^−11^	–	1.60 × 10^−12^	–
	1st cycle	6.02 × 10^−11^	1.21 × 10^−08^	9.68 × 10^−12^	1.95 × 10^−09^
	2nd cycle	7.22 × 10^−11^	7.35 × 10^−09^	1.51 × 10^−11^	1.54 × 10^−09^
	3rd cycle	7.49 × 10^−11^	5.12 × 10^−09^	8.96 × 10^−08^	6.12 × 10^−06^
	4th cycle	8.89 × 10^−07^	4.08 × 10^−05^	8.89 × 10^−07^	4.08 × 10^−05^
	5th cycle	5.46 × 10^−06^	1.88 × 10^−04^	5.49 × 10^−06^	1.89 × 10^−04^
RE	Non coating	3.88 × 10^−11^	–	3.27 × 10^−11^	–
	1st cycle	1.32 × 10^−11^	1.19 × 10^−08^	4.33 × 10^−10^	4.20 × 10^−08^
	2nd cycle	4.05 × 10^−10^	1.98 × 10^−08^	1.66 × 10^−10^	8.12 × 10^−09^
	3rd cycle	8.46 × 10^−06^	2.79 × 10^−04^	8.46 × 10^−06^	2.79 × 10^−04^
	4th cycle	3.68 × 10^−05^	9.20 × 10^−04^	7.02 × 10^−06^	1.76 × 10^−04^
	5th cycle	6.41 × 10^−05^	1.18 × 10^−03^	2.10 × 10^−05^	3.87 × 10^−04^

### Performance as strain sensor and Poisson’s ratio

**Figure 6:**
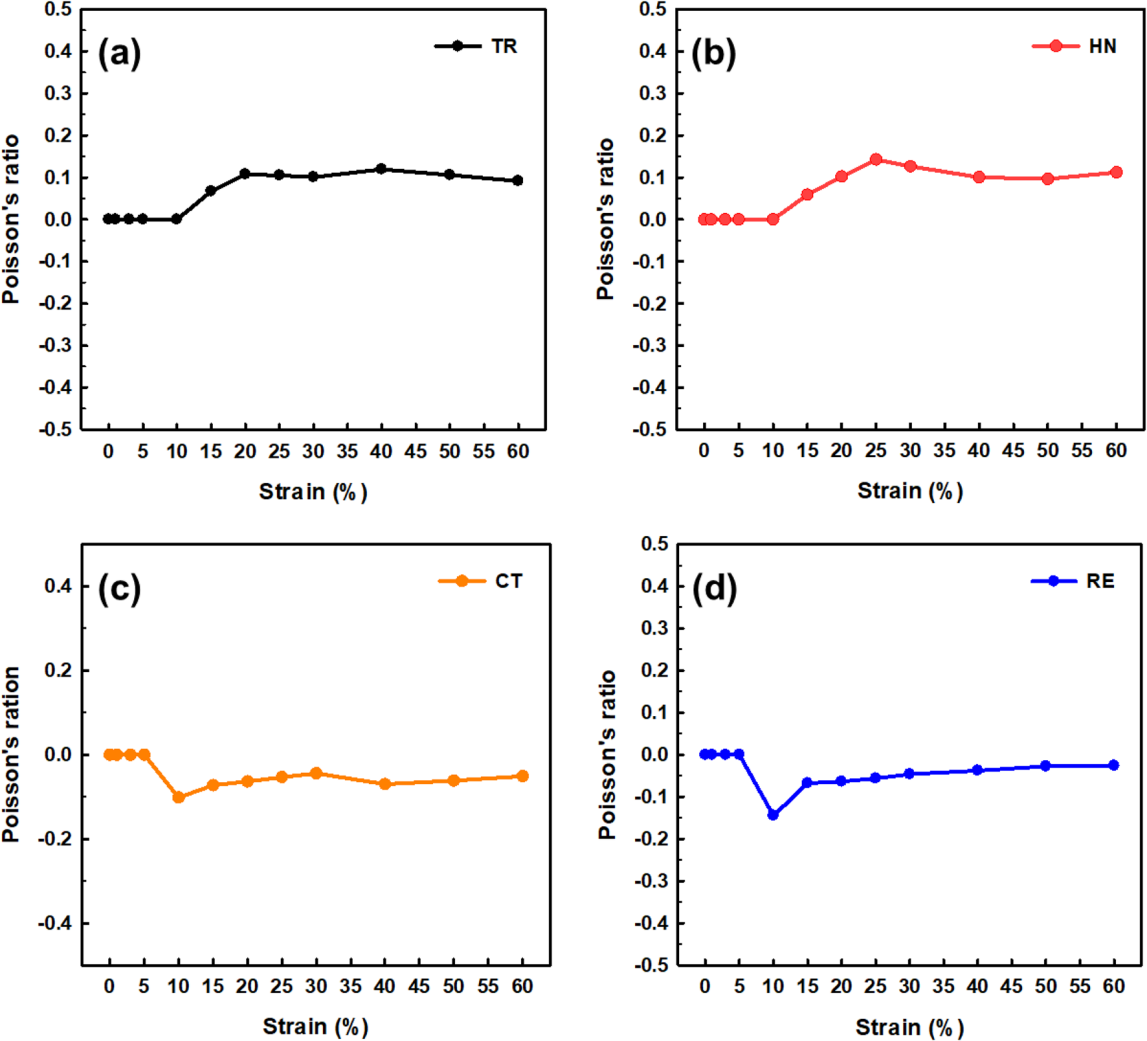
Poisson's ratio changes of auxetic lattice 4 type structures: (**a**) TR, (**b**) HN, (**c**) CT, and (**d**) RE.

For each 3D-printed auxetic lattice 4 type structure, Poisson's ratio was calculated by analyzing the images depicted under strain as a deformation property, and the rate of change of relative resistance during elongation was measured, as a strain sensor. Tables 5, 6, 7, 8 are a series of images showing the deformation behavior extending up to 60% over the gauge length for all 4 type structures, and Poisson's ratio was plotted from the square box (red line) to measure the changes in the repeating unit of each pattern. The two regular structures, TR and HN, exhibited a positive Poisson's ratio and showed a general characteristic of expanding transversally while axially compressed. CT and RE exhibited a negative Poisson's ratio, and they expanded transversally as they are axially stretched^47^. The auxetic structure is one of the metamaterials that obtain properties from the structure^48^, and all 4 type structures printed as designed exhibited a Poisson's ratios suitable for their characteristics. Figure 6 are graphs showing the Poisson's ratio extracted from each image by plotting it for each structure. The 4 type structures maintained either positive or negative Poisson's ratio until the 60% of strain, as shown in Fig. 6a–d. At large levels of uniaxial stretching, the auxetic structures experience nonlinear finite deformations aligned closely to the loading direction by huge loadings^49^. The TPU-based 4 type structures we designed were shown to exhibit stable structures at strains up to 60%.

**Table 5:**
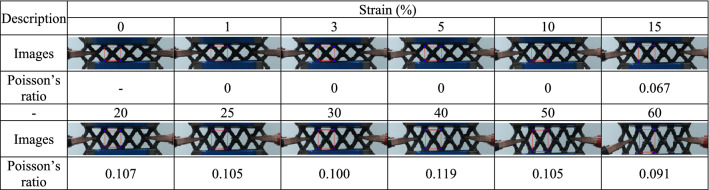
Strain images and Poisson’s ratio of auxetic lattice TR patterns up to 60% elongation.

**Table 6:**
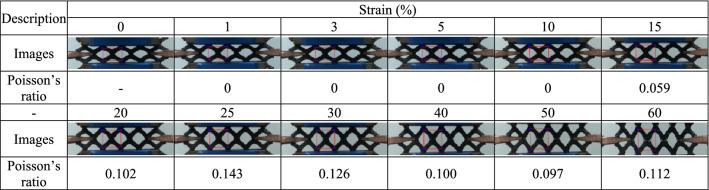
Strain images and Poisson’s ratio of auxetic lattice HN patterns up to 60% elongation.

**Table 7:**
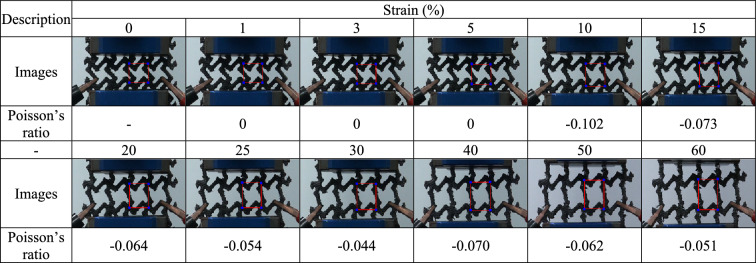
Strain images and Poisson’s ratio of auxetic lattice CT patterns up to 60% elongation.

**Table 8:**
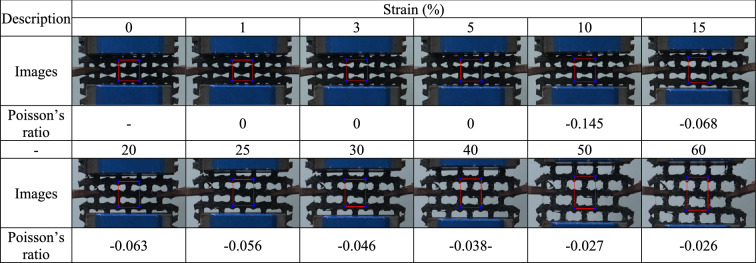
Strain images and Poisson’s ratio of auxetic lattice RE patterns up to 60% elongation.

**Figure 7:**
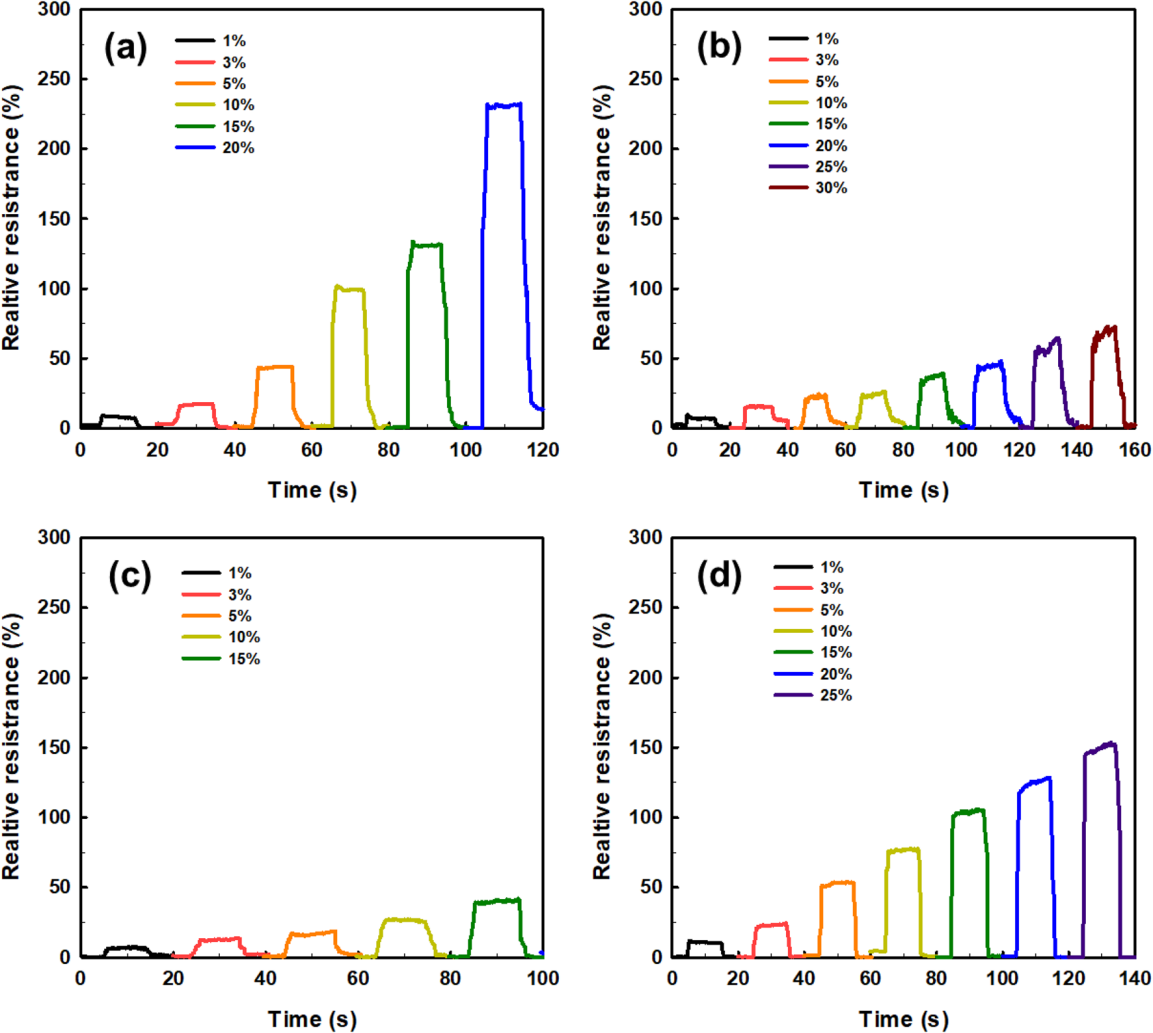
Relative resistance changes of auxetic lattice 4 type structures: (**a**) TR, (**b**) HN, (**c**) CT, and (**d**) RE.

Finally, the performance as strain sensors was compared with the TR and HN with positive Poisson's ratio and CT and RE with negative Poison's ratio, as shown in Fig. 7. For the measurement, the sensor was elongated for 20 s for a predetermined strain range and held for 10 s before it returned to its original position. After mounting the electrodes in the CD on each structure, the strain signal was connected to the source meter, and the change in the resistance was outputted to the computer through the signal.

Each sensor was reliably sensitive to a displacement of 1% for all structures, and the detection of this initial resistance change is the result of an efficient CWPU/Graphene water-based coating solution process. However, the limit of the graphene coating of TPU at 60% strain was different for each structure. TR and HN with positive Poisson's ratio detected changes in the resistance up to 20% and 30% strains, which are related to the durability of the coated CWPU and the magnitude of reversible deformation with each structural change. In fact, as shown in Tables 5 and 6, TR and HN show these results due to damage to the coating at the intersection of the structures at the elongation of 20% and 30%. CT and RE with a negative Poisson's ratio detected changes in the resistance up to 15% and 25% strains, and also microscopic damages at the intersection of these structures were confirmed as shown in Tables 7 and 8. In addition, CT seems to have a limit of 15% due to the greater reversible deformation during strain, further reinforcing this result. However, all structures transmitted signals very stably for a period of 10 s to respond. In applications, the sensors can reliably identify a constant strain by the change in resistance^50^.

**Figure 8:**
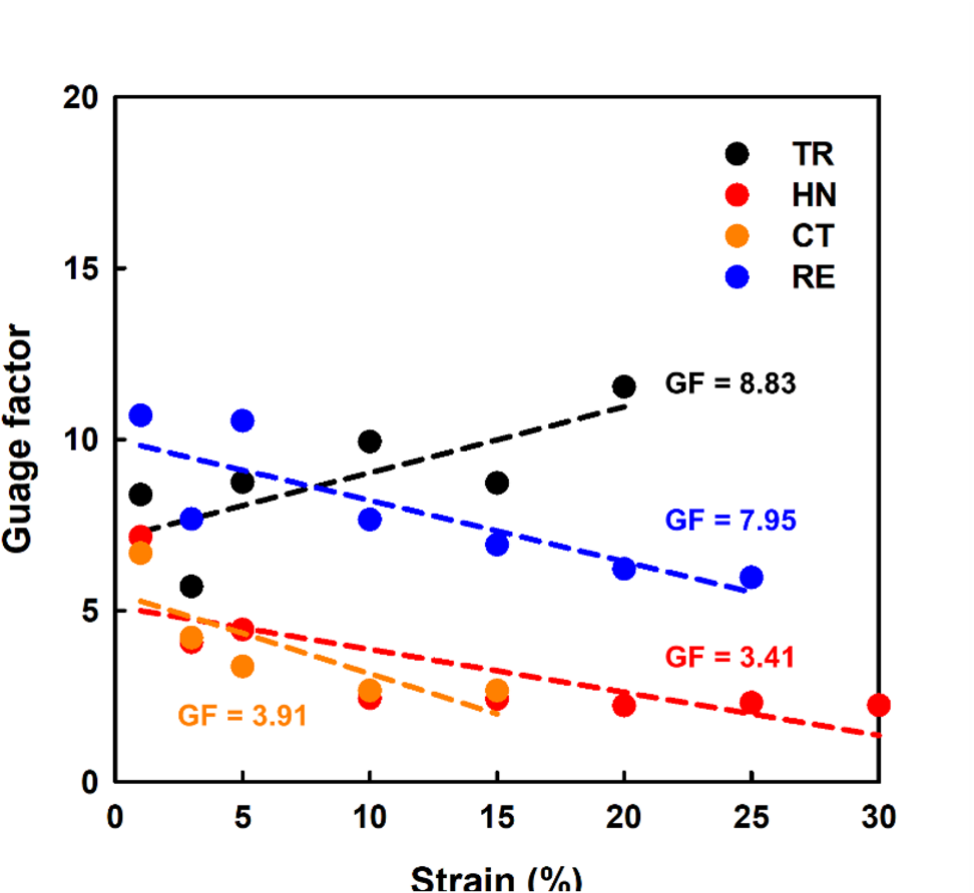
Variation in relative resistance *vs.* strain. Gauge factors are averaged obtained for each strain.

Figure 8 shows that TR, HN, CT, and RE exhibited Gauge Factors of 8.83, 3.41, 3.91, and 7.95, respectively, before reaching the maximum measurable strain. All of the calculated gauge factors were sufficient for use as strain sensors. And a sensor with a positive Poisson's ratio has better TR than HN. Also, as a sensor with a negative Poisson's ratio, the RE's performance is better. Although we only showed the design of 4 models for the strain sensor, it was shown that the 3D printed TPU materials stably coated with the CWPU/Graphene water-based solution could act as a sensor. Our results provide new opportunities for TPU-based auxetic lattice structures to be used in various electronic products using the dip-coating method.

## Conclusion

In conclusion, we 3D-printed TPU through FDM printing to generate two of the 4 types with positive and negative Poisson's ratio respectively and coated them with graphene to evaluate them as strain sensors. After coating the surface of the printed TPU with the CWPU/Graphene water-based solution more than 5 times and evaporating moisture, the graphene network was combined to form a stretchable material and a conductive coating film. In addition, the mechanical properties after coating 5 times improved the strength of all the structures. TR and HN with positive Poisson's ratio have resistance sensing ranges of 20% and 30%, respectively, while CT and RE with negative Poisson's ratio have 15% and 25%, respectively, and act as sensors. In particular, all of them can be used as flexible wearable sensors that identify various strain motions through linear and stable resistance changes over the entire range of each strain.
